# Low temperature induces embryonic diapause in the spider mite, *Eotetranychus smithi*

**DOI:** 10.1093/jis/14.1.68

**Published:** 2014-01-01

**Authors:** Tetsuo Gotoh, Yasunobu Kameyama

**Affiliations:** Laboratory of Applied Entomology and Zoology, Faculty of Agriculture, Ibaraki University, Ami, Ibaraki 300- 0393, Japan

**Keywords:** aging, life-history strategy, strawberry, overwintering

## Abstract

The spider mite, *Eotetranychus smithi* Pritchard & Baker (Acari: Tetranychidae), exhibits a facultative diapause that occurs at the egg stage. Diapause was induced by low temperatures alone (≤ 17.5°C) and averted by high temperatures (≥ 20°C). Photoperiod had little effect on diapause induction. This is the first example of temperature-induced diapause in spider mites. The diapause eggs became larger and darker (orange) than non- diapause eggs (white to pale yellow), suggesting that egg size and egg color are associated with diapause. When mites that were reared from eggs at 25°C and 16:8 L:D were transferred to 15°C and 16:8 L:D just after the start of the teleiochrysalis stage (the final molting stage before adulthood), all females laid non-diapause eggs during the first 30 days and then switched over to laying diapause eggs. The switch to diapause may be caused by the aging of mothers.

## Introduction

Many arthropods enter diapause to synchronize their activities to favorable times and to enhance their survival during unfavorable periods in the year (Friedrich 1984; Tauber et al. 1986 ; Danks 1987 , 2007; Friberg et al. 2011 ). Diapause induction is not a direct response to unsuitable environmental conditions but is determined in advance by environmental cues that herald the approach of an unfavorable season. In the temperate zones, photoperiods mainly regulate the timing of the onset of diapause, and temperatures can have a secondary effect by enhancing or inhibiting the induction of diapause by photoperiod ( Claret and Carton 1980 ; Tauber et al. 1986 ; Danks 1987 ). Nevertheless, in some arthropods, diapause induction is controlled entirely by temperature alone ( Danks 1987 ); high or low temperatures induce diapause at a single specific stage. Examples are the mosquito *Aedes vexans* (Friedrich 1984), the tobacco hornworm, *Manduca sexta* ( Cantelo 1974 ), the pipevine swallowtail, *Battus philenor* ( Sims and Shapiro 1983 ), and parasitoid wasps such as *Trioxys complanatus* ( Flint 1980 ) and *Lep- topiliana boulardi* ( Claret and Carton 1980 ; Carton and Claret 1982 ).

The spider mite, *Eotetranychus smithi* Pritchard & Baker (Acari: Tetranychidae), is a multivoltine species that infests various berries, roses, pears, and grapes in the United States, Brazil, and Asia, including China, Korea, and Japan ( Jeppson et al. 1975 ; Ehara and Gotoh 2009 ; Mendonça et al. 2011 ). *Eotetranychus smithi* is a potential pest of grape and strawberry in Japan, and sometimes outbreaks occur in greenhouse cultivated crops ( Itagaki 1998 ; Ehara and Gotoh 2009 ). The *E. smithi* Akitsu strain enters facultative diapause in the embryonic stage under 20°C/12 hr light and 10°C/12 hr dark conditions ( Ashihara 2001 ). More than 70% of the eggs laid at 15°C constant temperature and under a 10:14 L:D photoperiod were found to hatch (i.e., > 70% non-diapause eggs) ( Ashihara 2001 ).

These observations indicate that diapause induction is controlled by an appropriate combination of photoperiod and temperature. On the other hand, in the *E. smithi* Nagasaki strain, preliminary data suggest that low temperatures (15 and 17.5°C) induce egg diapause independently of photoperiod. The former diapause response may be adaptive for *E. smithi* inhabiting a strawberry greenhouse, where night temperatures are much lower than day temperatures. The winter temperature in a strawberry greenhouse is around 8°C at night, even with gas heating, and is around 25°C during the day (24-hr average is 15–17°C) under long-day conditions (16 hr light by artificial lighting). However, it is unknown whether *E. smithi* females lay non-diapause or diapause eggs under such conditions of fluctuating temperature and long-day photoperiod. If egg diapause of *E. smithi* is exclusively induced by low temperature, as in the latter case, all *E. smithi* females that underwent juvenile development under low temperatures (15–17°C) in a greenhouse might lay diapause eggs, and also adult females initially laying non-diapause eggs might, in response to low temperatures, switch to laying diapause eggs. Whether adult female mites are sensitive to the diapause-inducing low temperatures and can switch to laying diapause eggs needs to be clarified.

The purpose of the present study was to test the hypothesis that switchover from non- diapause to diapause status occurs in the adult stage of *E. smithi* . In the first experiment, eggs were kept and juveniles developed to adults under various combinations of photoperiod (16:8 L:D or 10:14 L:D) and temperature (15, 17.5, 20, or 25°C) to assess the effect of temperature and photoperiod on egg type laid (diapause or non-diapause eggs). In a second experiment, teleiochrysalis females (final immature resting stage) that had developed from egg onwards at 25°C and 16:8 L:D photoperiod were transferred to 15°C and a 16:8 L:D photoperiod, and subsequently the type was monitored of all eggs laid to clarify whether adult females can switch from producing non- diapause to diapause eggs, or vice versa.

## Materials and Methods

Several tens of *E. smithi* were collected originally from *Rosa multiflora* Thunb. (Rosales: Rosaceae) on 14 August 2007 at Kuchinotsu, Nagasaki, Japan (32°35’N; 130°10’E). Laboratory stocks were reared on leaf discs (ca. 16 cm2) of either mulberry, *Morus bombycis* Koidz. (Moraceae) (July to September), or strawberry, *Fragaria* x *ananassa* Duchesne (Rosaceae) (race ‘Tochi-Otome’; October to June), placed on water-saturated polyurethane mats in plastic dishes (9 cm diameter, 2 cm depth) at 25 ± 1°C, 16:8 L:D photoperiod, and 60–70% relative humidity.

To determine the effects of temperature and photoperiod on induction of the embryonic diapause of *E. smithi* , groups of 20 inseminated female adults obtained from stock cultures were transferred onto leaf discs (5 cm diameter) of strawberry and kept at 15, 17.5, 20, or 25°C and 10:14 L:D or 16:8 L:D. Females were allowed to lay eggs for 24 hr under each set of these eight regimes. Eggs obtained were reared until all individuals reached the teleiochrysalis stage (final molting stage before adulthood). Single teleiochrysalis females (n = 32–105) were individually placed on a strawberry leaf disc (1 cm diameter) with two male adults obtained from stock cultures for copulation. All eggs except one were removed per female, and the remaining eggs were observed for 30 days to determine the duration of the egg period under the various conditions. The diameter of a random selection of 20 of these remaining eggs was measured using a binocular stereomicroscope (SZ-45, Olympus, ( www.olympus-global.com ) equipped with an ocular micrometer (U-OSM, Olympus), and egg color was recorded. Eggs used in this experiment were laid on the third or fourth day after the start of oviposition, that is, on day 17, 15, 6, and 4 of adult life of females kept at 15, 17.5, 20 and 25°C, respectively. On the third or fourth day, oviposition ranged from 1 to 11 eggs/female/day, depending on temperatures.

To assess fecundity and longevity of females reared under the various conditions, females obtained from stock cultures were individually transferred onto strawberry leaf discs (1 cm diameter) and allowed to lay eggs for 24 hr at 15°C/10:14 L:D, 15°C/16:8 L:D, 17.5°C/16:8 L:D, or 25°C/16:8 L:D. On each leaf disc, one egg was left and reared until all individuals reached the teleiochrysalis stage. Two male adults from stock cultures were added to each teleiochrysalis female for copulation. Females were monitored, and eggs were counted daily. If a female had laid one or more eggs since the previous observation, she was transferred onto a new leaf disc (1 cm diameter) and this procedure was repeated until she died. Leaf discs with eggs were moved to the 25°C/16:8 L:D condition and kept there for 10 days to determine whether these eggs would hatch or not. Egg duration of non-diapause eggs ranged from three to five days when females had been reared and oviposited at 25°C/16:8 L:D (see Table 1 ).

**Table 1.**
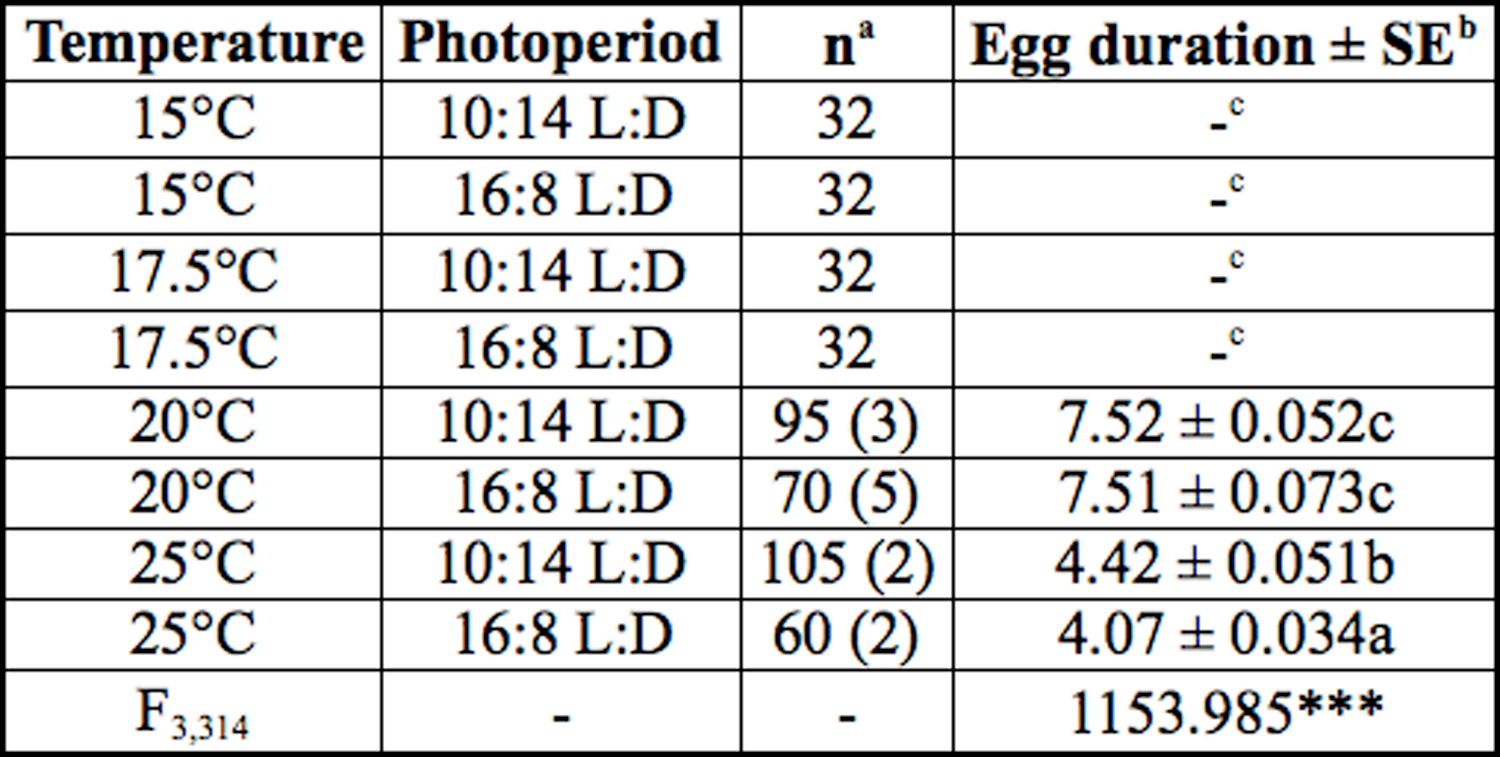
Effects of temperature and photoperiod on egg duration (days) of *Eotetranychus smithi.*

^a^ Number of eggs tested. As 2–5 unhatched eggs, shown in parentheses, were observed at 20 and 25°C, they were discarded from the calculation of egg duration.

^b^ Data analyzed using ANOVA; ***: *P* < 0.001. Values followed by the same letters are not significantly different (Tukey's HSD test, p>0.05).

^c^ Eggs did not hatch even if they were kept for > 30 days under each condition.

A second batch of 20 teleiochrysalis females that had undergone juvenile development under 25°C/16:8 L:D was transferred to 15°C/16:8 L:D (temperature-switch treatment). Again, two male adults were added for copulation, and eggs were counted daily, as described above. The transfer of adult females to a lower temperature (same photoperiod) may provide more insight in the mite’s developmental phase sensitivity to diapause- inducing stimuli.

Data on egg period, egg size, oviposition period, pre- and postoviposition periods, adult longevity, and total fecundity per female were analyzed using oneway analysis of variance (ANOVA) followed by Tukey’s honestly significant difference (HSD) test for multiple comparisons. Numbers of diapause eggs and non-diapause eggs obtained from the temperature-changed condition were compared using a χ2-test. These statistical analyses were performed using PASW ver. 18 for Windows ( SPSS 2009 ). To normalize the data and to achieve variance homogeneity prior to ANOVA, all values were ln-transformed ( Sokal and Rohlf 1995 ).

## Results

At 15 and 17.5°C, all females laid diapause eggs regardless of the photoperiod ( Table 1 ); no eggs hatched even if they were kept for more than 30 days at either of the various conditions. Mites that were maintained at temperatures of 20°C or higher laid non- diapause eggs, and their incubation periods were about eight and four days at 20 and 25°C, respectively ( Table 1 ). In conclusion, induction of diapause in *E. smithi* is temperature dependent.

Diapause eggs were larger than non-diapause eggs ( *P* < 0.05, Tukey’s HSD test; Table 2 ). Diapause eggs were orange, whereas non- diapause eggs were white to pale yellow. These secondary characteristics are useful for discriminating diapause and non-diapause eggs because they provide the diapause status of eggs without the need to wait for hatching.

**Table 2.**
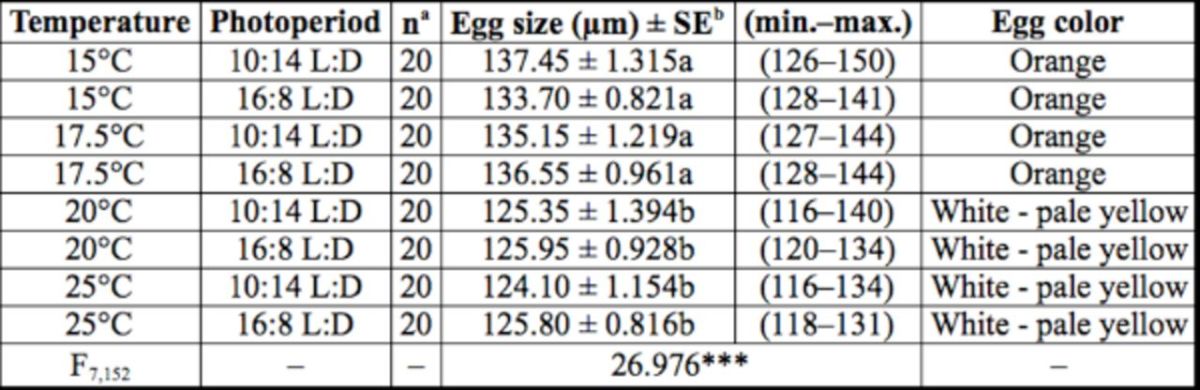
Effects of temperature and photoperiod on egg size and egg color of *Eotetranychus smithi.*

^a^ Number of eggs tested.

^b^ Data analyzed using ANOVA; ***: *P* < 0.001. Values followed by the same letters are not significantly different (Tukey's HSD test, *P* > 0.05).

**Figure 1.**
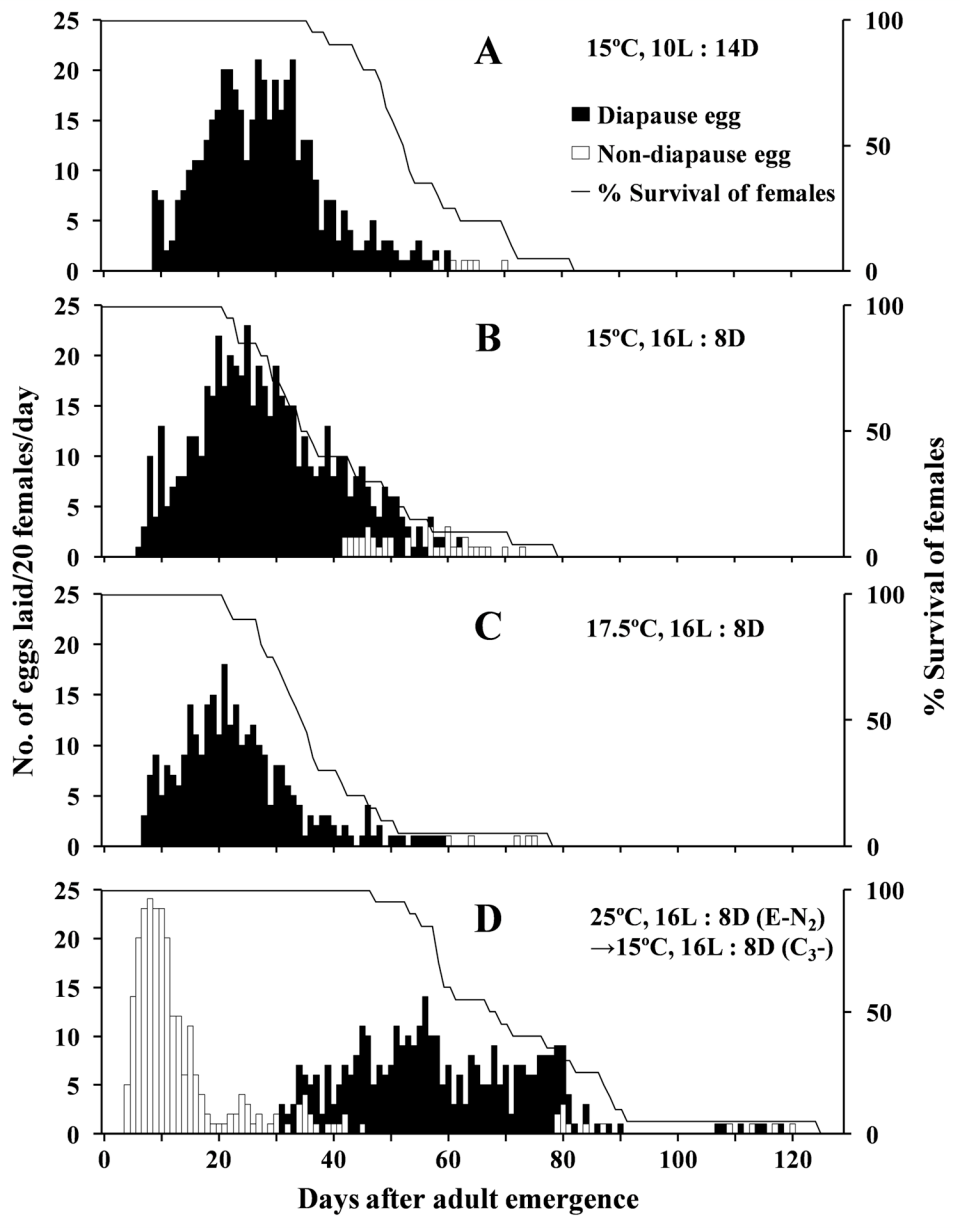
Age-specific female survival (%) and age-specific fecundity of females (n = 20) of *Eotetranychus smithi* under various sets of conditions. E, N2, and C3 indicate egg, deutonymph, and teleiochrysalis, respectively. See text for details. High quality figures are available online.

Most females that were held at 15 or 17.5°C deposited diapause eggs throughout their lives, but a few females (n = 1–4) began to lay non-diapause eggs near the end of their lives ( Figure 1A –C). By contrast, all females that had developed as juveniles under 25°C/16:8 L:D and were transferred to 15°C/16:8 L:D at the beginning of the teleiochrysalis stage laid non-diapause eggs during the first 30 days of their lives and then began to lay diapause eggs. Interestingly, a few females (n = 3) restarted to lay non-diapause eggs after 80 days ( Figure 1D ). The diapause eggs laid by females that had experienced the temperature switch were also orange and their average (±SE) size was 135.16 ± 1.127 µm (based on a sample of single eggs from 19 49- to 55-day- old adult females), which is not significantly different from the size of eggs laid at constantly low temperatures ( *P* = 0.16, F4,94 = 1.706).

Under constant temperature and photoperiodic conditions, both the oviposition period and total longevity were shortened with increasing temperature, but they were 1.5 to 3 times longer under the temperature-switch condition ( *P* < 0.05, Tukey’s HSD test; Table 3 ). Total fecundity under constant 17.5°C/16:8 L:D condition was significantly lower, and under constant 25°C/16:8 L:D condition it was significantly higher than fecundity under the other conditions. Under the temperature- switch treatment, females laid more diapause than non-diapause eggs (57 vs. 43%; *P* < 0.01, χ2 = 10.632).

**Table 3.**
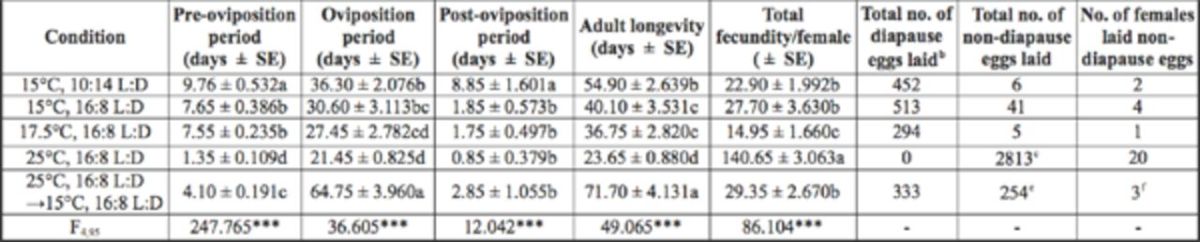
Effects of temperature and photoperiod on various reproductive parameters and number of eggs laid in *Eotetranychus smithi.* a

^a^ 20 females were tested at each condition. Data analyzed using ANOVA; ***: *P* < 0.001. Values followed by the same letters within a column are not significantly different (Tukey's HSD test, *P* > 0.05).

^b^ Unhatched orange eggs were regarded as diapause eggs.

^c^ All eggs were white to pale yellow.

^d^ Mites were transferred from 25°C to 15°C just after start of the teleiochrysalis stage.

^e^ Six unhatched eggs laid after 100 days of adult life were regarded as non-diapause eggs, because their color was white to pale yellow.

^f^ Females first laid non-diapause eggs, next laid diapause eggs, and then laid non-diapause eggs again.

## Discussion

The present study clearly showed that low temperatures alone (≤ 17.5°C) induce diapause in eggs of *E. smithi* independently of photoperiod. This is the first example of temperature-induced diapause in spider mites. Temperature-induced diapause is not rare in tropical arthropod species, where seasonal variation in daylength is very small; for example, the difference in daylength between summer and winter is only 7 min in Nairobi, Kenya ( Denlinger 1974 ; Danks 1987 ). In temperate zones, about 60% of female adults of the Japanese parasitoid wasp, *Ooencyrtus*

*nezarae* , entered reproductive diapause under 15°C/16:8 L:D ( Numata 1993 ), showing that low temperature alone can induce diapause for some adult females. Ashihara (2001) , who used the Akitsu (34°19’N) strain of *E. smithi* , showed that more than 70% of eggs laid under 15°C/10:14 L:D could hatch, whereas close to 100% of eggs laid under 20°C/12 hr light and 10°C/12 hr dark entered diapause. These findings clearly differed from the findings of our study, despite the close similarity of rearing conditions in the two studies (10°C gap between high and low temperatures). We feel confident about our findings because we confirmed the diapause status of the eggs at 15 and 17.5°C, both at 10:14 L:D and 16:8 L:D, both in the experiment on egg duration ( Table 1 ) and in the experiment on fecundity and longevity ( Figure 1A –C). Further studies are needed to explain this discrepancy.

The females of *E. smithi* that had first laid non-diapause eggs switched over to laying diapause eggs by day 30 of their adult life without any simultaneous changes in stimuli, such as temperature or photoperiod ( Figure 1D ). The switchover in egg type has been demonstrated in another spider mite, *Oligonychus ununguis* ( Akita 1971 ), in which females first laid non-diapause eggs and then switched to laying diapause eggs without any environmental stimuli. Such switchover of diapause status may be due to the effect of maternal aging on diapause induction ( Mousseau and Dingle 1991 ). In the blowfly, *Calliphora vicina* , non-diapause larvae developed from eggs that were laid on days 9, 11, or 18 by the adults kept at 20°C/18:6 L:D ( Nesin et al. 1995 ). By contrast, diapause- destined larvae were produced from eggs that were laid on days 24 or 31 by adults kept under the same conditions. The diapause larvae accounted for 28% of the total progeny produced on day 24 and for 44% of the total progeny produced on day 31 ( Nesin et al. 1995 ). Thus, aged females of *C. vicina* can produce some diapause-destined progeny even under constant temperature and long-day conditions. The switchover in diapause status may be adaptive for multivoltine species living in temperate zones. Females that experienced low temperatures can switch from non- diapause to diapause status, but females do (and should) not react quickly to low temperatures because of extreme fluctuations of autumn climate ( Figure 1D ). Females need to experience a threshold number of days of low temperatures to trigger the switch in diapause status of their eggs. Further studies are needed to determine how many cycles of low temperature are required for the switchover of diapause status in *E. smithi* .

## References

[R1] Akita Y . 1971 . Biological studies of the common conifer spider mite, *Oligonychus ununguis* Jacobi (Acarina: Tetranychidae) . Bulletin of the Government Forest Experiment Station 236 : 1 – 25 . (in Japanese)

[R2] Ashihara W . 2001 . Biology and seasonal occurrence of *Eotetranychus smithi* . Plant Protection 55 : 471 – 474 . ( in Japanese )

[R3] Cantelo WW . 1974 . Diapause in a tropical strain of the tobacco hornworm . Annals of the Entomological Society of America 67 : 828 – 830 .

[R4] Carton Y Claret J . 1982 . Adaptative significance of a temperature induced diapause in a cosmopolitan parasitoid of *Drosophila* . Ecological Entomology 7 : 239 – 247 .

[R5] Claret J Carton Y . 1980 . Diapause in a tropical species, *Cothonaspis boulardi* (parasitic Hymenoptera) . Oecologia 45 : 32 – 34 . 28310933 10.1007/BF00346703

[R6] Danks HV . 1987 . Insect Dormancy: an ecological perspective . Biological Survey Canada .

[R7] Danks HV . 2007 . The elements of seasonal adaptations in insects . Canadian Entomologist 139 : 1 – 44 .

[R8] Denlinger DL . 1974 . Diapause potential in tropical flesh flies . Nature 252 : 223 – 224 . 4418088 10.1038/252223a0

[R9] Ehara S Gotoh T , Editors. 2009 . Colored guide to the plant mites of Japan . Zenkoku- Noson-Kyoiku-Kyokai .

[R10] Flint ML . 1980 . Climatic ecotypes in *Trioxys complanatus* , a parasite of the spotted alfalfa aphid . Environmental Entomology 9 : 501 – 507 .

[R11] Friberg M Haugen IMA Dahlerus J Gotthard K Wiklund C . 2011 . Asymmetric life-history decisionmaking in butterfly larvae . Oecologia 165 : 301 – 310 . 20953962 10.1007/s00442-010-1804-0 PMC3021710

[R12] Friederich P . 1984 . Temperature-induced dormancy in laboratory and wild eggs of the floodwater mosquito *Aedes vexans* Meigen (Diptera: Culicidae) . Zeitschrift für Angewandte Zoologie 73 : 353 – 368 .

[R13] Itagaki N . 1998 . Spider mites observed on delaware grape in Shimane . Annual Report (1998) of Shimane Agricultural Experiment Station (Plant Protection Group) . ( in Japanese )

[R14] Jeppson LR Keifer HH Baker EW . 1975 . Mites injurious to economic plants . University of California Press .

[R15] Mendonça RS Navia D Diniz IR Flechtmann CHW. 2011 . South American spider mites: New hosts and localities . Journal of Insect Science 11 : 121 . Available online: www.insectscience.org/11.121 22224405 10.1673/031.011.12101 PMC3281375

[R16] Mousseau TA Dingle D . 1991 . Maternal effects in insect life histories . Annual Review of Entomology 36 : 511 – 534 .

[R17] Nesin AP Simonenko NP Numata H Chernysh SI . 1995 . Effects of photoperiod and parental age on the maternal induction of arval diapause in the blowfly, *Callipora**vicina* Robineau-Desvoidy (Diptera: Calliphoridae) . Applied Entomology and Zoology 30 : 351 – 356 .

[R18] Numata H . 1993 . Induction of adult diapause and of low and high reproductive states in a parasitoid wasp, *Ooencyrtus nezarae* , by photoperiod and temperature . Entomologia Experimentalis et Applicata 66 : 127 – 134 .

[R19] Sims SR Shapiro AM . 1983 . Pupal diapause in *Battus philenor* (Lepidoptera: Papilionidae) . Annals of the Entomological Society of America 76 : 407 – 412 .

[R20] Sokal RR Rohlf FJ . 1995 . Biometry . WH Freeman and Company.

[R21] SPSS . 2009 . Manual of PASW® Statistics Base 18 . SPSS Japan Inc . ( in Japanese )

[R22] Tauber MJ Tauber CA Masaki S. 1986 . Seasonal adaptations of insects. Oxford University Press .

